# A Rare Cause of Refractory Chronic Diarrhea and Cachexia: A Case Report

**Published:** 2020-02

**Authors:** Faten HADJ KACEM, Donia CHEBBI, Amal CHAKROUN, Nadia CHARFI, Dorra GHORBEL, Fatma MNIF, Mouna MNIF, Nabila REKIK, Mohamed ABID

**Affiliations:** Department of Endocrinology, Hedi Chaker Hospital, Sfax, Tunisia

**Keywords:** Neuroendocrine tumor, Somatostatin analogue, Vipoma, Secretory diarrhea, Tunisia

## Abstract

VIPoma is an unusual neuroendocrine neoplasm that autonomously secretes VIP. It is associated with secretory diarrhea and electrolyte disturbances. Herein we report a case of a male patient, who was hospitalized in the Department of Endocrinology in Hedi Chaker Hospital, Sfax, Tunisia. He presented VIPoma syndrome, with hepatic metastases at diagnosis. He had a history of chronic, watery diarrhea. He was dehydrated with many electrolytic disorders as hypokalemia, hyponatremia and metabolic acidosis. Abdominal CT scan showed a heterogeneous mass in the pancreatic head with multiple hepatic lesions. A high VIP hormone level was found. Histological study of a liver biopsy revealed hepatic metastases of neuroendocrine carcinoma. The patient received analogues of somatostatin and systemic chemotherapy, with a transient symptomatic relief. Sadly the patient was lost to follow-up.

## Introduction

VIPoma is a very rare disease with an incidence rate of 1 case per 10 million person-years (1). It is a neuroendocrine tumor causing watery diarrhea, hypokalemia and achlorydria due to secretion of vasoactive intestinal polypeptide (VIP) hormone. The histology of tumor and the dosage of VIP in a blood sample confirm the diagnosis. Somatostatin analogue is a simple and efficient treatment for diarrhea. Curative treatment with surgery could be proposed for localized disease. In case of unresectable or progressive disease, systemic chemotherapy may be needed.

We present the case of a patient diagnosed in our service with VIPoma, extensive hepatic metastases and severe life-threatening diarrhea.

## Case report

A 39 year old man who had no particular medical history, was hospitalized in the Endocrinology Department in Hedi Chaker, Sfax, Tunisia. He presented for 2 years chronic watery diarrhea (7 to 10 times/day) without vomiting, abdominal pain, or rectorrhagia. The diarrhea was unresponsive to normal antidiarrheal agents. He had anorexia and unencrypted weight loss. The abdominal ultrasound showed a metastatic nodular liver. It was then completed with liver biopsy puncture. Histology and immunohistochemistry showed: hepatic metastases of neuroendocrine carcinoma with cells strongly expressing chromogranin A and weakly AFP.

Informed consent was taken from the patient before the study.

Contrasted computed tomography (CT) of the abdomen showed a heterogeneous mass in the head of the pancreas, with multiple hypodense hepatic nodules measuring between 11 and 38 mm.

Colonoscopy was normal and microscopy showed ulcerated bulbitis. Chemotherapy was indicated but the patient refused it. Six months later, the patient had developed polyuro polydipsic syndrome, diarrhea persisted, and his condition worsened, he had therefore benefited of a cure of chemotherapy: vinblastine 450 mg per day and cysplatine 150 mg per day. He was also treated with Sandostatine and Minirin 0.3ml three times a day. The symptoms were initially improved and then recurred.

Therefore, the patient was transferred to our service. He was asthenic, bedridden and had signs of intra and extra cellular dehydration. His blood pressure was 90/50 mmHg and his pulse was 80 bpm. His weight was 39kg with a body mass index 14 kg per m^2^. His physical exam showed no other anomalies. He had clinical euthyroidism and eucorticism.

Biological findings were: hyponatrémia (119 mmol/l) kypokalémia (2.31mmol) hypocalcémia (1.96 mmol/l) and metabolic acidosis. His kaliuresis was 32.55mmol/24h. The plasma vasoactive intestinal peptide (VIP) level was 216 ng/ml. The diagnosis retained was vasoactive intestinal polypeptide-secreting tumor (VIPoma) with liver metastases.

The patient was urgently rehydrated with potassic supplementation through a supraclavicular central venous catheter and treated with Minirin 0.3 ml* 2 per day, calcium gluconate 16g/24 hand somatostatin analogues. The evolution was marked by the improvement of potassium levels, the degression of polyuro polydipsic syndrome which was due to hypokalemia and the persistence of diarrhea which became less abundant.

During hospitalization, the patient developed a pulmonary embolism treated with curative doses of anticoagulants, and a catheter-related bloodstream infection, requiring the removal of the catheter and the use of broad-spectrum antibiotics.

The option of palliative surgery was discussed but eliminated because of the poor general health condition of the patient. He was then transferred to the carcinology department to receive a chemotherapy cure. The patient was lost to follow-up.

## Discussion

VIPoma also called Verner-Morrison syndrome, and the WDHA syndrome (watery diarrhea, hypokalemia, and hypochlorhydria or achlorhydria), is a very rare neuroendocrine tumor that autonomously secretes VIP. It occurs in 1 per 10 million individuals per year (1).

VIPomas consist of less than 10% of all pancreatic islet cell tumors, and almost all of them (90%) originate from the pancreas in adults, mostly in the tail. Whereas some extra-pancreatic tissues can secrete VIP factor, especially in pediatrics such as bronchus, colon, and liver. (2)

Because of its rarity, the diagnosis of VIPoma is often missed, and distant metastasis are often observed at the time of diagnosis (80%) (3).

Liver is the most usual metastasis site of VIPomas, as the case of our patient, but other metastasis sites are also reported, such as lymph nodes, kidneys and lungs (2).

VIPoma is described as “pancreatic cholera”, characterized by large-volume watery diarrhea without steatorrhea and hypokalemic acidosis as a result of potassium and bicarbonate loss in the stool.

Potassium and bicarbonate wasting result in hypokalemic non-anion gap metabolic acidosis. Our patient had obvious watery diarrhea that did not stop after symptomatic treatment, and the level of potassium in the blood was very low with metabolic acidosis. He also had polyuropolydipsic syndrome as a result of the hypokalemia.

High VIP hormone levels are always found in VIPomas, and VIP levels greater than 200 pg/mL are required to confirm the diagnosis(4), which was also true in the present case (VIP=216pg/ml).

Most VIPomas are voluminous, making them easily identified by CT/MRI, octreotide scan, and endoscopic ultrasound (4).

To retain the diagnosis of VIPoma, typical clinical symptoms are required, with an elevated plasma VIP level. CT and ultrasonography can localize the tumor and its metastasis. Octreoscan scintigraphy is an extremely helpful radiological intervention, although it is rarely used (5,6).

In terms of histopathology, VIPomas are neuroendocrine tumors.

Immunohistochemically, they stain positively for VIP, chromogranin A, synaptophysin, somatostatin, neuron specific enolase and cytokeratin. In our case the tumor was strongly expressing chromogranin A and weakly AFP. VIPoma is difficult to treat in the vast majority of cases.

The first step in the treatment of these patients is the vigilant correction and maintenance of electrolytes. If a tumor has been identified, surgical resection is the gold standard.

In the case of metastatic VIPomas, surgery is also the first choice, especially in the case of liver metastasis, which is the case of our patient. In fact, after removing the primary tumor and hepatic metastatic lesions, the patient’s symptoms are often relieved and disease-free survival is prolonged as well (7). Unfortunately, the poor general condition of our patient did not allow surgery. In the case of inoperable patients, as the case of our patient, non-surgical treatments are indicated for symptomatic relief of diarrhea.

Somatostatin and its analogs are the first choice for controlling VIPoma symptoms as suggested by North American Neuroendocrine Tumor Society (NANETS) consensus guidelines (8).

They are used to control diarrhea, reduce VIP levels. They also have anti-proliferative properties stabilizing tumor growth.

Chemotherapy must also be considered in unresectable metastatic diseases. It has been used in few series with varying success rates. In our case, the patient received both somatostatin analogs and chemotherapy, with a transient relief of the symptoms.

VIPomas are very rare tumors, often diagnosed at a metastatic stage. We have to suspect them when confronted with severe chronic diarrhea. Treatment options include surgery, chemotherapy, and somatostatin analogs, but prospective randomized trials are not available to guide management because of the rarity of these tumors.

## Ethical considerations

Ethical issues (Including plagiarism, informed consent, misconduct, data fabrication and/or falsification, double publication and/or submission, redundancy, etc.) have been completely observed by the authors.
